# Ageism, negative attitudes, and competing co-morbidities – why older adults may not seek care for restricting back pain: a qualitative study

**DOI:** 10.1186/s12877-015-0042-z

**Published:** 2015-04-08

**Authors:** Una E Makris, Robin T Higashi, Emily G Marks, Liana Fraenkel, Joanna E M Sale, Thomas M Gill, M Carrington Reid

**Affiliations:** 1 Department of Internal Medicine, UT Southwestern Medical Center, 5323 Harry Hines Blvd, Dallas, TX 75390-9169 USA; 2 Department of Clinical Sciences, UT Southwestern Medical Center, Dallas, TX USA; 3 Department of Veterans Affairs, Dallas, TX USA; 4 Department of Medicine, Yale School of Medicine, New Haven, CT USA; 5 Department of Veterans Affairs, West Haven, CT USA; 6 Musculoskeletal Health and Outcomes Research, Li Ka Shing Knowledge Institute, St. Michael’s Hospital, Toronto, Canada; 7 Institute of Health Policy, Management & Evaluation, University of Toronto, Toronto, Canada; 8 Division of Geriatrics and Palliative Medicine, Weill Cornell Medical College, New York, NY USA

**Keywords:** Aging, Back pain, Qualitative research, Musculoskeletal conditions

## Abstract

**Background:**

Back pain, the most common type of pain reported by older adults, is often undertreated for reasons that are poorly understood, especially in minority populations. The objective of this study was to understand older adults’ beliefs and perspectives regarding care-seeking for restricting back pain (back pain that restricts activity).

**Methods:**

We used data from a diverse sample of 93 older adults (median age 83) who reported restricting back pain during the past 3 months. A semi-structured discussion guide was used in 23 individual interviews and 16 focus groups to prompt participants to share experiences, beliefs, and attitudes about managing restricting back pain. Transcripts were analyzed in an iterative process to develop thematic categories.

**Results:**

Three themes for why older adults may not seek care for restricting back pain were identified: (1) beliefs about the age-related inevitability of restricting back pain, (2) negative attitudes toward medication and/or surgery, and (3) perceived importance of restricting back pain relative to other comorbidities. No new themes emerged in the more diverse focus groups.

**Conclusions:**

Illness perceptions (including pain-related beliefs), and interactions with providers may influence older adults’ willingness to seek care for restricting back pain. These results highlight opportunities to improve the care for older adults with restricting back pain.

## Background

Back pain is the most common type of musculoskeletal pain reported by older adults [1,2], and the second most common complaint for which patients visit a primary care doctor’s office [3-5]. Each year, more than $100 billion is spent in the US on back pain [6], and costs are expected to rise considerably with our rapidly aging population [7]. Pain management and treatment are particularly challenging because clinical guidelines do not currently exist for back pain in older adults; moreover, guidelines for back pain in younger populations fail to account for decision-making complexities that occur commonly in older populations, such as multiple comorbid conditions [8], poly-pharmacy, frailty, and fragmented social support systems [9].

Prior literature suggests that back pain in older adults is neither inevitable nor should it go untreated [9-14]. A substantial body of literature points to ageism as a potential reason for under-treatment of pain in older adults [15,16]. By ‘ageism’ we mean the overt and subtle ways in which older adults may be unfairly assessed and treated by medical providers, e.g., as physically or mentally disabled or unworthy of treatment, simply because of their advanced age. We know that ageism or negative age stereotypes have been implicated in unfavorable outcomes [17-19], although this has not been explored to a great extent in the back pain literature specifically. Despite efforts to combat negative perceptions of older adults, the negative impact of ageism on older adults’ physical and psychological health persists [20-22].

Although several studies have shown that back pain among older adults is frequently undertreated [9,23-25], few studies have explored *how* and *why* this is the case [11,26]. This paper addresses both questions by qualitatively assessing the experience of back pain from the perspective of older adults [11,27], and documenting how the patient-provider relationship may impact the decision to seek care—both of which could contribute to under-treatment of back pain in older adults. Qualitative methods using semi-structured interviews and focus groups elicit a thorough understanding of older adults’ perspectives regarding pain, care-seeking and decision-making strategies by allowing participants to elaborate on their feelings and rationale, rather than focusing solely on quantified end results [28,29].

Older adults and those of racial/ethnic minorities are at higher risk of being under-treated for pain [30-32]. Studies have shown that minority patients with pain are more likely to report greater activity limitations, severe pain, and functional impairments compared with non-Hispanic whites [33,34]. The sources of pain disparities among racial/ethnic minorities are complex and occur at the provider level (e.g. lack of education), system level (e.g. lack of access to pain medications), and patient level (e.g. cultural beliefs about pain) [35]. Despite this knowledge, prior studies of back pain among older adults have included mostly non-Hispanic White populations and have largely focused on patients actively seeking treatment [32,36,37]. Thus it is unclear whether findings from these studies also pertain to racially/ethnically diverse samples [38], and those who are not actively seeking treatment (e.g. individuals who reported pain to their physician while seeking care for some other health problems, or are simply living with back pain) [38-40].

Despite a few qualitative studies of older adults’ experiences with back pain [11,26,41], there is limited understanding of *why* older adults sometimes choose not to seek care. The objective of this research was to understand the experiences of older adults who report back pain severe enough to restrict activity, hereafter referred to as restricting back pain. In this paper we focus on specific beliefs and attitudes in a racially diverse population of older adults, and how they may impact care-seeking for restricting back pain.

## Methods

This study employed systematic qualitative data collection methods [42-47], consistent with qualitative research procedures [48,49]. Qualitative methods were chosen to solicit feedback from a racially diverse sample of older adults (age ≥65 years) who had experienced restricting back pain during the past three months [50]. Participants were excluded if they were non-English speaking or unable to actively participate in an interview or focus group setting.

The researchers used three different recruitment strategies: first, participants were recruited from the Yale Precipitating Events Project (PEP), as previously described [29]. Twenty-three semi-structured interviews were conducted by the principal investigator in participants’ homes, with each interview lasting an average of 45 minutes (range of 20–60 minutes). The semi-structured interview guide was developed initially based on the purpose of the study, existing literature and clinical experience. The guide was revised after the first three interviews to make the language easier to understand by older adults. The semi-structured interview guide prompted participants to discuss their experiences with restricting back pain, including their beliefs and behaviors related to pain management (“What coping mechanisms are you using or have you used for your back pain?”, “Was it helpful?”), the impact of back pain (“How does your back pain affect you physically?” “How does your back pain affect you emotionally?”), problems with other medical conditions (e.g. “How do your other medical conditions affect the way you experience back pain, and vice versa?”), and experiences interacting with doctors (“Do you talk to your doctors about your back pain?”, “What has your doctor told you about your back pain?”, “What can your doctor do better to help you with your back pain?”)

Next, the researchers recruited participants for focus groups to assess whether the feedback from interview participants was shared by other populations in the community. Of the 16 total focus groups, 9 were conducted in Connecticut and 7 in New York City (NYC); focus groups lasted between 30–60 minutes and were comprised of 3–7 participants each. The Connecticut-based focus groups were conducted first, following an educational session on arthritis management. To enhance the racial/ethnic diversity of the sample, the researchers subsequently recruited participants from NYC senior centers that primarily served Black and Hispanic clients in order to confirm or verify consistency or discrepancies in emerging themes. The structured focus group guide was developed using the same questions as the interview guide with the exception of language used to engage participants in responding to and discussing other participants’ experiences of back pain and their ideas about self-care and care-seeking.

Like the CT participants, the NYC participants were 65 years or older, community-living, and reported restricting back pain during the last three months. Recruitment from NYC senior centers was facilitated by individual senior center directors, as previously described [38,51,52]. Each focus group had approximately 3–7 participants to facilitate contribution from all participants and to capture the diversity of their experiences, attitudes, and preferences [53]. Focus groups were conducted in English by the lead author (UM) or by a research team member. For interviews and focus groups, recruitment ended once no new themes related to our study purpose were emerging.

### Data analysis

All interviews and focus groups were audio recorded; audio files were transcribed verbatim and data were analyzed in NVivo 9.0 (QSR International, AUS), a software program that allows researchers to code, organize, sort, and report qualitative data. Investigators evaluated the first six interview transcripts using an inductive, text-driven approach to qualitative thematic-analysis [46,54,55]. We developed a coding scheme in which meaningful statements were identified from transcripts and assigned codes; thereafter codes were re-evaluated and revised in an iterative process. All transcripts were analyzed by a team of three coders and led by the principal investigator. In order to establish coding consistency within the team, four interviews were selected at random to be analyzed and discussed by all team members until consensus was reached. The final coding scheme and code definitions were then organized in a qualitative codebook that served as a guide for all coders and subsequent coding activities. Each transcript was assigned to two coders in a matrix that ensured that all coders were paired evenly across transcripts for balanced analysis. Routine team meetings were held to review progress during analysis [42,43]; discrepancies were discussed during these meetings until consensus was reached.

Following the CT focus groups the researchers conducted an interim analysis of findings, which revealed a high level of thematic consistency with the PEP interview data. The final analysis of data following the seven NYC focus groups (with diverse older adults) determined that no new themes had emerged from the NYC sample as compared with the CT samples. All data were de-identified prior to analysis. This study was approved by the Yale University and UT Southwestern Medical Center’s Institutional Review Boards.

## Results

Participants’ demographic characteristics are summarized in Table 1. Twenty-three interviews (n = 23) and 16 focus groups were conducted (n = 70 participants), for a total of 93 participants. Among all participants, 68% were female, the median age was 83 years, 17% had less than a high school education, and more than half lived alone. Participants in the CT focus groups and one-on-one interviews were racially and ethnically similar (primarily non-Hispanic White), while seven of the nine (44%) focus groups in NYC included predominately Black and/or Hispanic participants. Over half of the study participants (n = 51, 55%) reported restricting back pain for 10 years or longer and nearly a quarter (n = 24, 26%) for 5–9 years; 76 (82%) of the participants had tried or were currently using an oral pain medication, 18 (19%) had tried topical therapies, 69 (74%) reported using a combination of non-pharmacologic management strategies (e.g. physical therapy, yoga, and exercise), and 54 (58%) reported using hot/cold modalities for pain relief.

**Table 1:** **Interview and focus group participant characteristics**

	Interview participants	Focus group participants
Number of participants	n = 23	n = 70
Age		
<80 years	0 (0%)	36 (52%)
80- 84 years	7 (30)	11 (16%)
≥85 years	16 (70%)	20 (29%)
Gender		
Female	13 (57%)	50 (71%)
Male	10 (43%)	20 (29%)
Ethnicity		
Hispanic	0 (0%)	8 (11%)
Non-Hispanic	23 (100%)	57 (81%)
Unknown	0 (0%)	5 (7%)
Race		
African American/Black	2 (9%)	26 (37%)
Caucasian/White	21 (91%)	36 (51%)
Other/Multiracial	0 (0%)	7 (10%)
Unknown	0 (0%)	1 (1%)
Education level		
Less than 12 years	5 (22%)	11 (16%)
Living Arrangements		
Living Alone	12 (52%)	37 (53%)

Analysis of the data revealed several potential reasons underlying participants’ motivation for avoidance of seeking care. Although participants were not directly prompted to address patient-provider communication, narratives reveal that the nature of interactions with providers figured centrally in participants’ attitudes toward care-seeking strategies. We identified three themes that emerged as potentially related to participants’ decisions to seek future medical care or not: (1) participant and perceived provider beliefs about the inevitability of restricting back pain in later life (voiced by 74% of interview participants and 75% focus groups); (2) participants’ negative attitudes toward medication and/or surgery (74% interviews and 94% focus groups); and (3) the relative importance of restricting back pain versus other comorbidities (96% interviews and 94% focus groups). All three themes emerged from both the interviews and the focus groups.

Note that, although the discussion guide did not directly ask participants whether these experiences would prevent them from seeking care for restricting back pain in the future, participant remarks (e.g., “I’ll just go through life the way it is”) and the tone with which they describe their experiences (e.g., “the heck with it”) suggest disinterest in future engagements with providers.

### Participant-reported beliefs about age-related inevitability of restricting back pain

Several participants reported that restricting back pain was a normal, commonly occurring part of the aging process. This notion of the inevitability of restricting back pain with older age was reported both as a personal belief and as a message often communicated by providers. These feelings were expressed in phrases such as: “I think it’s part of aging,” (NYC focus group participant), “I figured/assumed everyone has [pain]” (PEP interview participant) and “my body’s worn out,” (CT and NYC focus group participants), “The older I get, I think the worse it’d be… it last longer, and it hurt more,” (NYC focus group participant).

Commonly, these participants reported that their providers either dismissed or minimized their restricting back pain, often with ageist statements. These interactions appeared to either reinforce already held perceptions that back pain was inevitable, and/or limit motivation to seek further pain care in the future, as shown in the following participant narratives.

I’ll tell you what the doctor thinks: “you’re 93 years old!” I see that all the time when I go to the office. Like everything is taken very lightly. So, I’d rather be my own doctor. (PEP interview participant)

I went to him [doctor] and I said, “Look I can’t walk. What am I supposed to do?” He says, “How old are you?” I said, “I’m close to 90.” “What do you expect? You’re an old man.” (NYC focus group participant)

Thus, dismissive or minimizing comments by providers can serve to inform or reinforce older adults’ beliefs that back pain is directly related to old age, or perhaps, that providers have nothing more to offer.

### Negative attitudes toward pharmacologic and surgical interventions

Several participants described negative attitudes toward pharmacologic and surgical treatments.

Participants were animated in their remarks about the perceived lack of efficacy, adverse effects, or fear of becoming addicted to medications.

You could use a world of medication; [the back pain] is still there. (NYC focus group participant)

One of the [providers] gave me Percocet. I took exactly one and I threw the rest of them away. It was the most horrible experience of my life. I was in la-la land. (CT focus group participant)

If they say I must do it, then I’ll take one…first of all it makes me groggy and all I want to do is sleep and I don’t want to get hooked on it so I don’t want to take now. (NYC focus group participant)

Well, medicine is okay but I don’t want to become addicted… Once you become an addict of medicine…the stronger they get…You take [morphine] and you fall asleep and when you wake up you still got the pain, you still got the issues. (NYC focus group participant)

Many participants expressed wariness of taking multiple medications, reported fear of taking “yet another” medication, or worried about how the medication might negatively interact with their existing medications and potentially cause more harm than benefit.

They always want to give me medicine. I don’t want medicine! Because I don’t think it helps any…I don’t want another medication. (PEP interview participant)

I’m taking so much medication, I don’t like to take too much stuff….So I let the pain go away, come and go the way you please and that is it. (PEP interview participant)

…they give you this pill for that, that pill have a side effect, then when you go back…they going to give you pills for the [side] effects. Then that pill is going to have another [side] effect, and that what messes up your whole metabolism. (NYC focus group participant)

While some participants expressed negative attitudes toward medications, others expressed dissatisfaction with their providers. That is, participants frequently said that their provider was not listening to their concerns or was only willing to use medication rather than discussing or considering alternatives.

They give me more pills but I don’t want more pills. “Why don’t you try this?” “No I don’t want, I really don’t want more pills”… I want to be able to correct it somehow but not with pills. (PEP interview participant)

I feel like they want to give you medicine and I’m against that. (NYC focus group participant)

That's the thing. They don't tell you much. They'd rather give you medication. (NYC focus group participant)

[The orthopedic specialist] saw me, took some x-rays…I don’t think I even had therapy. He gave me medication for pain… The last couple of months I’d see him for five minutes and that’s it. He’d have me, once in a while, walk- but there was no real treatment other than pain killers. (PEP interview participant)

The impact of negative patient-provider interactions was evident when participants described providers giving mixed or conflicting information about treatment options, not communicating adequately, or not soliciting participants’ feedback in establishing treatment goals. Many participants felt that providers either had nothing to offer or were not willing to offer anything new, which possibly served as a deterrent to future care-seeking.

So when you go [to the doctor], you come back knowing less than you did when you went. Because they don’t have time to explain to you the whys … when you find out more about what’s going on with your body, I think you can accept it better than to wonder. (PEP interview participant)

I’m not gaining anything [new]. I go and I listen and nothing happens… I’d love to have some options. (PEP interview participant)

Some interactions with providers were specifically focused on the perception that surgery would be the next or the only treatment proposed, and this perception led some participants to avoid seeking care. Participants’ comments about surgery were mostly negative, and ranged from a fear of proposed or presumed surgical options to actual suboptimal outcomes among surgical patients.

If it bothered me, I would like to have it treated but not operated on…I don’t know how to put it. Fear, I think it’s fear mostly. (PEP interview participant)

I figure it’s something I’m going to have to probably live with. I don’t have any faith that surgery would be of any reasonable help. (PEP interview participant)

That's a scary thing. Because I know a few people that had to have surgery…Because they never get over it…They always have problems. (NYC focus group participant)

They left it up to me to decide whether I would have [the operation] or not, and, of course, I didn’t have it. There’s always a risk with everything…I didn’t know if it would make me better or make it worse. I figured if I could tolerate what I have now, I’ll just go through life with the way it is. (PEP interview participant)

I was planning to get surgery, oh, maybe about 11 years ago and the more I thought about it and the more I talked to the doctor—there are only three things that could happen: 1) you’re great like a new person, 2) you’re the same, not a bit of change, and the other is, 3) you’re worse! So I say, two out of three is not good, *the hell with it*! So I canceled the operation! *(emphasis original)* (PEP interview participant)

Poor patient-provider communication resulted in discordant treatment goals, expectations, and definitions of “success”, and appeared to contribute to participants’ negative overall perceptions of surgical treatment options. One participant who had had back surgery remarked:

[The surgeon] told me [the back surgery] was a success … he said “it worked out” from the X-ray, that it looks like it’s going to be successful, but the pain…that’s what to me, what I would say is successful, if I didn’t have any more pain. As long as I have pain, it was not successful! (PEP interview participant)

### Relative importance of restricting back pain versus other comorbidities

Many participants encountered uncertainty in managing multiple comorbid conditions. This may have been exacerbated by the fact that older adults often receive care from a variety of medical specialists (e.g. cardiologists, endocrinologists, rheumatologists), each of whom may be more focused on treating “their” condition. Further, participants sought care for conditions they perceived to have higher priority than their restricting back pain.

I mean, I have to watch my diet. And I do have to see the doctor regularly for my kidney condition. So I don't worry about the back pain. I just worry about having a fall or something. (PEP interview participant)

My concentration at this point is my diabetes. I’ve had that for almost 30 years. And that has presented problems along the way…they know more about that than what I’m going through with my back. (PEP interview participant)

I am having back pain…not only for months, for years. My doctor told me that they are only patching me up because I have other problems; prostate, liver, heart, and all different problems…so we got to live with all that. (NYC focus group participant)

It’s my knees I gotta worry about. Both my knees… wow, they hurt. (PEP interview participant)

While the majority of participants alluded to or discussed having challenging interactions with their provider, a few stated that they continued to experience restricting back pain but were willing to continue seeking care because of a positive relationship with their provider. As one participant said, “He’s a wonderful person, compassionate, and I’m grateful…if there’s something I could do…I’m willing and I’m able to make myself available.” (PEP interview participant). This scenario was not reported frequently, perhaps because the study eligibility criteria included participants who had experienced restricting back pain within the last three months.

## Discussion

This study contributes qualitative data from a racially/ethnically diverse group of older adults with restricting back pain and describes their beliefs about the inevitability of pain in older age, a common fear of surgery and polypharmacy, and challenges in managing restricting back pain in the context of multiple comorbid conditions. Our results suggest that patient-provider interactions, which underscored these discussions, can serve as powerful deterrents to future care-seeking. This is significant because, while patient-provider communication has been identified as both a significant barrier and facilitator to care in general adult populations [56-58], this research highlights aspects of the experience of restricting back pain from the older adult’s perspective.

By documenting the accounts of older adults with restricting back pain and their associated rationales for seeking or not seeking care, this study identifies opportunities for clinical intervention. For example, enhanced medical education to recognize and combat ageist beliefs and behaviors may help to reduce the under-treatment of restricting back pain in older adults. Future research should focus on quantifying both older adults’ and providers’ attitudes and beliefs about ageism and developing curricula/training programs that help providers learn how to respond to older adults’ feedback about their pain experiences, beliefs, and treatment goals [59-63]. Understanding how enhanced patient-provider relationships ultimately impact patient-reported or performance-based outcomes has yet to be determined among older adults with restricting back pain [64-67] and therefore warrants future attention.

Negative attitudes towards medication and surgery were provided mostly by participants who felt they had received inadequate or conflicting information from providers. These narratives also revealed a misalignment of priorities among participants versus providers, with participants feeling that provider recommendations did not adequately address their concerns. This finding underscores the importance of focusing on and enhancing patient-provider communication to develop a shared understanding of treatment goals and priorities and offers insight as to potential avenues for integrated interventions [63,68-70].

As demonstrated in our study, restricting back pain rarely occurred in isolation as most participants reported the presence of multiple chronic conditions. It is unclear how providers approach and evaluate an older adult’s experience of back pain relative to other comorbidities [8]. Studies have demonstrated that pain is consistently undertreated among older adults [23-25], and among minorities in particular [30-32]; however, these studies did not address *why* so many older adults continue to suffer with back pain. It is possible that providers undertreat pain because of its subjective nature, and instead focus on more acute problems or quantifiable assessments such as disease biomarkers or scales of severity [71]. Given the lack of data about the reasons underlying the under-treatment of back pain among older adults, qualitative studies such as this one-- pointing toward specific barriers (and some that are potentially modifiable) to seeking care-- are essential for identifying future targets for intervention. Further research is needed to evaluate why back pain remains undertreated and what multi-component interventions are most likely to succeed for older adults with multiple comorbidities.

The importance of patient-provider interactions and their impact on treatment outcomes and care-seeking has been well-documented in the field of mental health using the term ‘therapeutic alliance’ [64,65]. A recent qualitative study of older community-dwelling adults identified several factors contributing to their decisions to seek professional care, including: an aversion to vague age-related statements and an appreciation of diagnoses as professional validation of their concerns [16]. Likewise, our research highlights the importance of establishing an effective partnership, including the need to improve providers’ communication skills and willingness to engage older adults in their own care toward establishing shared treatment goals [10,72-74]. Educational curricula for providers should emphasize the potential influence of providers’ and older adults’ beliefs, biases, and illness perceptions on the patient-provider relationship [16]. Moreover, future studies should assess whether and how therapeutic alliance specifically ranks among other known factors that influence care-seeking [26,75] using a mixed-methods approach.

While membership in a minority group has been found to be a risk factor for the under-treatment of pain, prior research on back pain has been largely limited to persons who currently seek care and has often focused exclusively on non-Hispanic Whites [38]. We specifically broadened our sample from CT to NYC to enhance diversity. However, we did not have the resources to interview non-English speaking older adults. The research presented here enhances our understanding of how diverse populations experience back pain outside of the clinical setting by including older adults who may or may not have been engaged in care at the time of participation. This qualitative research is hypothesis generating and generalizing our findings to broader populations (for example, including non-English speakers) should be done with caution.

Limitations of this study include potential survival bias which may be inevitable in studies focused on older adults. Focus groups, while including Hispanic/Latino participants, were conducted in English only, which prevented Spanish-speaking only members of the community centers in NYC from taking part in the research. Older adults of different race/ethnicities who do not speak English may experience restricting back pain differently than those who speak English [35]. We acknowledge that the discussion guide and methodology used do not allow us to draw conclusions or generalizations about racial/ethnic groups. Further, there are likely socio-economic and other variables, that we did not assess/measure, that may contribute to whether older adults seek medical care. Focus groups were comprised of older adults who reported restricting back pain during the past three months and who were ambulatory enough to travel to a senior center, thereby excluding participants who were non-ambulatory and/or home-bound. This could have resulted in a sample whose pain was more manageable at the time of the focus group. We also acknowledge that clients who attend senior centers belong to a particular, sometimes self-selected, group who are able to engage in social events/ community activities. Still, our results from individual interviews, conducted in participants’ homes, were consistent with those from the focus groups. Lastly, we also acknowledge that the participants in our sample had engaged in care at some point and were reflecting on those experiences. We cannot be assured that the themes identified in this paper will directly result in the participant not seeking future care. However, the content and tone of the responses strongly suggest that the participants were dissatisfied with a clinical encounter and unlikely to return to the same provider for the same issue.

## Conclusions

By illustrating why older adults may not seek back pain-related care, this study contributes valuable information toward identifying potential opportunities to improve the quality of care for this growing population. When speaking about their experiences interacting with providers, older adults talked about the quality of interactions as a factor in decision-making about potential future care-seeking. Our findings suggest that older adults’ care-seeking behaviors and their attitudes toward providers and potential treatments for restricting back pain may be improved by enhanced patient-provider communication that includes eliciting goals of therapy and understanding older adults’ beliefs about and priorities for pain management strategies. Providers, in turn, might benefit from clinical guidance regarding the treatment of back pain specifically in older adult populations that recognizes the impact of ageist myths and assumptions and providers’ focus on medical and surgical interventions. Ultimately, by understanding why older adults may not seek care for restricting back pain, we may begin to uncover areas that can be improved upon, and thereby minimize the burden of undertreated back pain in older adults.

## References

[1] Leveille SG, Zhang Y, McMullen W, Kelly-Hayes M, Felson DT (2005). Sex differences in musculoskeletal pain in older adults. Pain.

[2] Podichetty VK, Mazanec DJ, Biscup RS (2003). Chronic non-malignant musculoskeletal pain in older adults: clinical issues and opioid intervention. Postgrad Med J.

[3] Deyo RA, Mirza SK, Martin BI (2006). Back pain prevalence and visit rates: estimates from U.S. national surveys, 2002. Spine (Phila Pa 1976).

[4] Deyo RA, Phillips WR (1996). Low back pain. A primary care challenge. Spine.

[5] Hart LG, Deyo RA, Cherkin DC (1995). Physician office visits for low back pain. Frequency, clinical evaluation, and treatment patterns from a U.S. national survey. Spine.

[6] Katz JN (2006). Lumbar disc disorders and low-back pain: socioeconomic factors and consequences. J Bone Joint Surg Am..

[7] Hoy D, March L, Brooks P, Blyth F, Woolf A, Bain C (2014). The global burden of low back pain: estimates from the Global Burden of Disease 2010 study. Ann Rheum Dis.

[8] Tinetti ME, Fried TR, Boyd CM (2012). Designing health care for the most common chronic condition–multimorbidity. JAMA.

[9] Reid MC, Bennett DA, Chen WG, Eldadah BA, Farrar JT, Ferrell B (2011). Improving the pharmacologic management of pain in older adults: identifying the research gaps and methods to address them. Pain Med.

[10] Cayea D, Perera S, Weiner DK (2006). Chronic low back pain in older adults: What physicians know, what they think they know, and what they should be taught. J Am Geriatr Soc.

[11] Lyons KJ, Salsbury SA, Hondras MA, Jones ME, Andresen AA, Goertz CM (2013). Perspectives of older adults on co-management of low back pain by doctors of chiropractic and family medicine physicians: a focus group study. BMC Complement Altern Med..

[12] Morone NE, Greco CM, Rollman BL, Moore CG, Lane B, Morrow L (2012). The design and methods of the aging successfully with pain study. Contemp Clin Trials.

[13] Westrom KK, Maiers MJ, Evans RL, Bronfort G. Individualized chiropractic and integrative care for low back pain: the design of a randomized clinical trial using a mixed-methods approach Trials. 2010;11. doi:10.1186/1745-6215-11-24. 10.1186/1745-6215-11-24 PMC2841162 20210996

[14] Thielke S, Sale J, Reid C (2012). Are These 4 Pain Myths Complicating Care?. J Fam Practice.

[15] Brown D (2004). A literature review exploring how healthcare professionals contribute to the assessment and control of postoperative pain in older people. J Clin Nurs.

[16] Clarke A, Martin D, Jones D, Schofield P, Anthony G, McNamee P (2014). "I Try and Smile, I Try and Be Cheery, I Try Not to Be Pushy. I Try to Say 'I'm Here for Help' but I Leave Feeling… Worried": A Qualitative Study of Perceptions of Interactions with Health Professionals by Community-Based Older Adults with Chronic Pain. PLoS One.

[17] Hausdorff JM, Levy BR, Wei JY (1999). The power of ageism on physical function of older persons: reversibility of age-related gait changes. J Am Geriatr Soc.

[18] Levy BR, Banaji MR. Implicit ageism. Ageism: Stereotyping and prejudice against older persons. 2002. p. 49–75.

[19] Levy BR, Pilver CE, Pietrzak RH (2014). Lower prevalence of psychiatric conditions when negative age stereotypes are resisted. Soc Sci Med..

[20] Butler RN (1969). Age-Ism: Another Form of Bigotry. Gerontologist.

[21] Higashi RT, Tillack AA, Steinman M, Harper M, Johnston CB (2012). Elder care as "frustrating" and "boring": understanding the persistence of negative attitudes toward older patients among physicians-in-training. J Aging Stud.

[22] Phelan A (2011). Socially constructing older people: examining discourses which can shape nurses’ understanding and practice. J Adv Nurs.

[23] Barber JB, Gibson SJ (2009). Treatment of chronic non-malignant pain in the elderly: safety considerations. Drug Saf.

[24] Collett B, O'Mahoney S, Schofield P, Closs SJ, Potter J (2007). The assessment of pain in older people. Clin Med (London, England).

[25] Tse MMY, Chan BSH (2004). Knowledge and attitudes in pain management: Hong Kong nurses' perspective. J Pain Palliat Care Pharmacother.

[26] Morone NE, Lynch CS, Greco CM, Tindle HA, Weiner DK (2008). "I felt like a new person." the effects of mindfulness meditation on older adults with chronic pain: qualitative narrative analysis of diary entries. J Pain.

[27] Goertz CM, Salsbury SA, Vining RD, Long CR, Andresen AA, Jones ME et al. Collaborative Care for Older Adults with low back pain by family medicine physicians and doctors of chiropractic (COCOA): study protocol for a randomized controlled trial Trials. 2013;14. doi:10.1186/1745-6215-14-18. 10.1186/1745-6215-14-18 PMC3557195 23324133

[28] Pope C, Mays N (1995). Research the parts other methods cannot reach: an introduction to qualitative methods in health and health services research. BMJ..

[29] Makris UE, Melhado TV, Lee SC, Hamann HA, Walke LM, Gill TM (2014). Illness Representations of Restricting Back Pain: The Older Person’s Perspective. Pain Med.

[30] Hadjistavropoulos T, Herr K, Turk DC, Fine PG, Dworkin RH, Helme R (2007). An interdisciplinary expert consensus statement on assessment of pain in older persons. Clin J Pain.

[31] Hanlon JT, Backonja M, Weiner D, Argoff C (2009). Evolving pharmacological management of persistent pain in older persons. Pain Med.

[32] Shavers VL, Bakos A, Sheppard VB (2010). Race, ethnicity, and pain among the U.S. adult population. J Health Care Poor Underserved.

[33] McIlvane JM, Baker TA, Mingo CA, Haley WE (2008). Are behavioral interventions for arthritis effective with minorities? Addressing racial and ethnic diversity in disability and rehabilitation. Arthritis Rheum.

[34] Reyes-Gibby CC, Aday LA, Todd KH, Cleeland CS, Anderson KO (2007). Pain in aging community-dwelling adults in the United States: non-Hispanic whites, non-Hispanic blacks, and Hispanics. J Pain.

[35] Green C, Anderson K, Baker T, Campbell LC, Decker S, Fillingim R (2003). The unequal burden of pain: confronting racial and ethnic disparities in pain. Pain Med.

[36] Anderson KO, Green CR, Payne R (2009). Racial and ethnic disparities in pain: causes and consequences of unequal care. J Pain.

[37] Stubbs D, Krebs E, Bair M, Damush T, Wu J, Sutherland J (2010). Sex Differences in Pain and Pain-Related Disability among Primary Care Patients with Chronic Musculoskeletal Pain. Pain Med.

[38] Townley S, Papaleontiou M, Amanfo L, Henderson CR, Pillemer K, Beissner K (2010). Preparing to implement a self-management program for back pain in new york city senior centers: what do prospective consumers think?. Pain Med.

[39] Saper RB, Sherman KJ, Cullum-Dugan D, Davis RB, Phillips RS, Culpepper L (2009). Yoga for chronic low back pain in a predominantly minority population: a pilot randomized controlled trial. Altern Ther Health Med.

[40] Song J, Chang HJ, Tirodkar M, Chang RW, Manheim LM, Dunlop DD (2007). Racial/ethnic differences in activities of daily living disability in older adults with arthritis: a longitudinal study. Arthritis Rheum.

[41] Bunzli S, Watkins R, Smith A, Schutze R, O'Sullivan P: Lives on Hold: A Qualitative Synthesis Exploring the Experience of Chronic Low-back Pain. *Clin J Pain* 2013. 10.1097/AJP.0b013e31827a6dd8 23370072

[42] Bradley EH, Curry LA, Devers KJ (2007). Qualitative data analysis for health services research: developing taxonomy, themes, and theory. Health Serv Res.

[43] Cohen DJ, Crabtree BF (2008). Evaluative criteria for qualitative research in health care: controversies and recommendations. Ann Fam Med.

[44] Mays N, Pope C (1995). Qualitative research: Observational methods in health care settings. BMJ.

[45] Creswell J, Klassen A, Plano Clark V, Smith K. Best Practices for Mixed Methods Research in the Health Sciences: National Institutes of Health, Office of Behavioral and Social Sciences Research. 2011.

[46] Priest H, Roberts P, Woods L (2002). An overview of three different approaches to the interpretation of qualitative data. Part 1: Theoretical issues. Nurse Res.

[47] Woods L, Priest H, Roberts P (2002). An overview of three different approaches to the interpretation of qualitative data. Part 2: Practical illustrations. Nurse Res.

[48] Sandelowski M (2000). Whatever happened to qualitative description?. Res Nurs Health.

[49] Sandelowski M (2010). What's in a name? Qualitative description revisited. Res Nurs Health.

[50] Makris UE, Fraenkel L, Han L, Leo-Summers L, Gill TM (2011). Epidemiology of restricting back pain in community-living older persons. J Am Geriatr Soc.

[51] Parker S, Vasquez R, Kahoe E, Henderson CR, Pillemer K, Robbins L (2011). A comparison of the arthritis foundation self-help program across three race/ethnicity groups. Ethn Dis.

[52] Reid MC, Chen E, Parker S, Henderson C, Pillemer K (2014). Measuring the Value of Program Adaptation: A Comparative Effectiveness Study of the Standard and a Culturally Adapted Version of the Arthritis Self-Help Program. HSS J.

[53] Pope C, Ziebland S, Mays N (2000). Qualitative research in health care. Analysing qualitative data. BMJ.

[54] Miles M, Huberman AM (1994). Qualitative Data Analysis: An Expanded Sourcebook.

[55] Boyatzis RE (1998). Transforming qualitative information: thematic analysis and code development.

[56] Briggs AM, Slater H, Bunzli S, Jordan JE, Davies SJ, Smith AJ (2012). Consumers' experiences of back pain in rural Western Australia: access to information and services, and self-management behaviours. BMC Health Serv Res..

[57] Corbett M, Foster NE, Ong BN (2007). Living with low back pain-Stories of hope and despair. Soc Sci Med.

[58] Snelgrove S, Liossi C (2009). An interpretative phenomenological analysis of living with chronic low back pain. Br J Health Psychol.

[59] Cherkin D, Deyo RA, Berg AO, Bergman JJ, Lishner DM (1991). Evaluation of a physician education intervention to improve primary care for low-back pain. I. Impact on physicians. Spine.

[60] Klein BJ, Radecki RT, Foris MP, Feil EI, Hickey ME (2000). Bridging the gap between science and practice in managing low back pain. A comprehensive spine care system in a health maintenance organization setting. Spine.

[61] Slater H, Briggs AM, Smith AJ, Bunzli S, Davies SJ, Quintner JL. Implementing Evidence-Informed Policy into Practice for Health Care Professionals Managing People with Low Back Pain in Australian Rural Settings: A Preliminary Prospective Single-Cohort Study. Pain Med. 2014. doi:10.1111/pme.12351. 10.1111/pme.12351 24433536

[62] Watt-Watson J, Murinson BB (2013). Current challenges in pain education. Pain Manag.

[63] Makris UE, Abrams RC, Gurland B, Reid MC (2014). Management of persistent pain in the older patient: a clinical review. JAMA.

[64] Ferreira PH, Ferreira ML, Maher CG, Refshauge KM, Latimer J, Adams RD (2013). The therapeutic alliance between clinicians and patients predicts outcome in chronic low back pain. Phys Ther.

[65] Fuentes J, Armijo-Olivo S, Funabashi M, Miciak M, Dick B, Warren S (2014). Enhanced therapeutic alliance modulates pain intensity and muscle pain sensitivity in patients with chronic low back pain: an experimental controlled study. Phys Ther.

[66] Harman K, Macrae M, Vallis M, Bassett R (2014). Working with people to make changes: a behavioural change approach used in chronic low back pain rehabilitation. Physiother Can.

[67] Pransky G, Borkan JM, Young AE, Cherkin DC (2011). Are we making progress?: the tenth international forum for primary care research on low back pain. Spine (Phila Pa 1976).

[68] Chou RAS, Stanos SP, Rosenquist RW (2009). Nonsurgical interventional therapies for low back pain: A review of the evidence for an American Pain Society clinical practice guideline. Spine.

[69] de Stampa M, Vedel I, Bergman H, Novella J-L, Lechowski L, Ankri J (2012). Opening the Black Box of Clinical Collaboration in Integrated Care Models for Frail, Elderly Patients. Gerontologist.

[70] Teh CF, Karp JF, Kleinman A, Reynolds CF, Weiner DK, Cleary PD (2009). Older People's Experiences of Patient-Centered Treatment for Chronic Pain: A Qualitative Study Pain Medicine. Pain Medicine.

[71] Ambrosino N, Serradori M (2006). Determining the cause of dyspnoea: linguistic and biological descriptors. Chron Respir Dis.

[72] Diachun L, Van Bussel L, Hansen KT, Charise A, Rieder MJ (2010). "But I see old people everywhere": dispelling the myth that eldercare is learned in nongeriatric clerkships. Acad Med..

[73] Eskildsen MA, Flacker J (2009). A Multimodal Aging and Dying Course for First-Year Medical Students Improves Knowledge and Attitudes. J Am Geriatr Soc.

[74] Darlow B, Dowell A, Baxter GD, Mathieson F, Perry M, Dean S (2013). The Enduring Impact of What Clinicians Say to People With Low Back Pain. Ann Fam Med.

[75] Hopton A, Thomas K, MacPherson H (2013). The acceptability of acupuncture for low back pain: a qualitative study of patient's experiences nested within a randomised controlled trial. PLoS One.

